# Discovery of a 29-Gene Panel in Peripheral Blood Mononuclear Cells for the Detection of Colorectal Cancer and Adenomas Using High Throughput Real-Time PCR

**DOI:** 10.1371/journal.pone.0123904

**Published:** 2015-04-13

**Authors:** Laura Ciarloni, Sahar Hosseinian, Sylvain Monnier-Benoit, Natsuko Imaizumi, Gian Dorta, Curzio Ruegg, On behalf of the DGNP-COL-0310 Study Group

**Affiliations:** 1 Diagnoplex SA, Epalinges, Switzerland; 2 Novigenix SA, Epalinges, Switzerland; 3 National Center for Competence in Research (NCCR), Molecular Oncology, Swiss Institute for Experimental Cancer Research (ISREC)-Ecole Polytechnique Fédérale de Lausanne (EPFL), Lausanne, Switzerland; 4 Department of Gastroenterology and Hepatology, Centre Hospitalier Universitaire Vaudois (CHUV) and University of Lausanne, Lausanne, Switzerland; 5 Department of Medicine, Faculty of Science, University of Fribourg, Fribourg, Switzerland; IRCCS Istituto Oncologico Giovanni Paolo II, ITALY

## Abstract

Colorectal cancer (CRC) is the second leading cause of cancer-related death in developed countries. Early detection of CRC leads to decreased CRC mortality. A blood-based CRC screening test is highly desirable due to limited invasiveness and high acceptance rate among patients compared to currently used fecal occult blood testing and colonoscopy. Here we describe the discovery and validation of a 29-gene panel in peripheral blood mononuclear cells (PBMC) for the detection of CRC and adenomatous polyps (AP). Blood samples were prospectively collected from a multicenter, case-control clinical study. First, we profiled 93 samples with 667 candidate and 3 reference genes by high throughput real-time PCR (OpenArray system). After analysis, 160 genes were retained and tested again on 51 additional samples. Low expressed and unstable genes were discarded resulting in a final dataset of 144 samples profiled with 140 genes. To define which genes, alone or in combinations had the highest potential to discriminate AP and/or CRC from controls, data were analyzed by a combination of univariate and multivariate methods. A list of 29 potentially discriminant genes was compiled and evaluated for its predictive accuracy by penalized logistic regression and bootstrap. This method discriminated AP >1cm and CRC from controls with a sensitivity of 59% and 75%, respectively, with 91% specificity. The behavior of the 29-gene panel was validated with a LightCycler 480 real-time PCR platform, commonly adopted by clinical laboratories. In this work we identified a 29-gene panel expressed in PBMC that can be used for developing a novel minimally-invasive test for accurate detection of AP and CRC using a standard real-time PCR platform.

## Introduction

Colorectal cancer (CRC) is the third most common cancer and second leading cause of cancer-related death among men and women in Europe [1]. Importantly, CRC is often curable, when diagnosed at early stages. Moreover, detection and removal of adenomatous polyps (AP) prevents CRC formation and decreases mortality due to CRC. Several countries have already adopted screening modalities for CRC and clinical practice guidelines recommend that average risk individuals begin regular screening at 50 years of age [2, 3]

Colonoscopy is the “gold standard” for AP and CRC diagnosis, however it is not the preferred method for mass screening because of its cost, invasiveness, low compliance and limited accessibility. Currently recommended non-invasive methods for mass screening include immunochemical and guaiac fecal occult blood testing (iFOBT, gFOBT). Yet, compliance with fecal tests is still suboptimal in countries with an FOBT screening program [4, 5, 6]. Therefore, there is still a large unmet need calling for a non- or minimally-invasive, compliant, cost-effective and accurate screening test to detect AP and CRC at early stages. A blood-based screening test is highly attractive due to its minimal invasiveness and high acceptance among patients. In particular we and others have reported signatures derived from peripheral blood mononuclear cells (PBMC) gene expression profiles associated with digestive [7, 8, 9, 10], breast [11], renal [12, 13], pulmonary [14] and bladder cancers [15]. These tests are conceptually different from classical tumor biomarker tests, as they are based on the detection of the host response to tumor-derived signals [16, 17] rather than on markers originating from the tumor itself.

When searching for differences in gene expression and identification of RNA transcripts to be subsequently used as potential biomarkers, commonly used methods include microarray-based DNA hybridization platforms or RNA-based sequencing techniques [18, 19]. Although powerful, these methods are complex, time consuming and expensive, and generate high volume of data that require specialized bioinformatics tools and competencies for their analysis. Moreover, identified genes of interest require further validation by more accurate and sensitive methods such real-time qPCR, before they can be translated into clinically useful tests [20]. In alternative to these techniques, high throughput real-time qPCR platforms demonstrated to perform well when candidate gene selection was driven by solid scientific evidence, in spite of the fact that they allow the analysis of only a fraction of the transcriptome [21]. Importantly, biomarker discoveries based on qPCR platforms have the major advantage that a further validation step in view of their clinical use is not required. Furthermore, they are substantially less expensive than whole genome approaches, allowing the analysis of a larger sample set and thus increasing the statistical power of the study.

Here we report the discovery and characterization of a 29-gene panel in PBMC for the detection of colorectal adenomas and carcinomas using a nanoliter high throughput qPCR platform (OpenArray) [22]. To this purpose we used samples prospectively collected from a multicenter, case-control study in which patients were referred for colonoscopy or scheduled for surgery for CRC removal. We also demonstrated that the gene panel could be easily transferred and implemented into a medical laboratory-friendly assay, which is a key step in the development of a new cost-effective, simple blood-based colorectal cancer screening test.

## Material and Methods

### Patients

A case-control study (DGNP-COL-0310), including three South Korean and six Swiss centers which enrolled 1665 subjects older than 50 years that were referred for colonoscopy by general practitioner or were scheduled for surgery, was conducted from June 2010 to April 2013. The study was specifically conceived and designed for the development and validation of a new test for CRC screening. The biomarker discovery phase took place during the first half of patient recruitment. For this purpose, a subset of 144 subjects, allocated to control, CRC and AP groups (Table 1), was randomly selected, and used for gene expression profiling by high throughput qPCR.

**Table 1:** Characteristics of the study population.

	Subjects (n)	Age median *(25%-75%)*	Men
**Total**	144	61 *(55–68)*	56%
**Controls**	50	59 *(53–66)*	44%
**Adenomas ≥ 1cm**	46	61 *(56–67)*	61%
**CRC**	48	64 *(56–68)*	65%
Stage I	12	-	-
Stage II	12	-	-
Stage III	12	-	-
Stage IV	12	-	-

Subjects had no first-degree family history of CRC or a known CRC predisposition, previous history of polyps or cancer including CRC, no hepatobiliary, genitourinary, autoimmune and inflammatory disorders, including inflammatory bowel diseases, infectious diseases and fever within 4 weeks before colonoscopy. Chronic diseases common in the old population, such as diabetes, hypertension, hypercholesterolemia, heart failure, were not considered as exclusion criteria. A control subject was defined as an individual without any past and present history of colorectal lesions or diseases (e.g. small adenomas, hyperplastic polyps, cancer). The AP group included subjects diagnosed with an adenoma larger than 1 cm, based on the endoscopic measurement. The CRC group included patients with carcinoma at all four TNM stages. Final diagnosis was based on colonoscopy and histopathological evaluation.

### Ethical approval

The study protocol (DGNP-COL-0310) was approved by the competent review boards and ethics committees for research on human subjects of Canton Bern, Switzerland (Kantonale Etikkommission Berne, No KEK 139/10), Canton St. Gallen, Switzerland (Ethikkommission des Kantons St. Gallen, No. EKSG 10/091/1B), Canton Vaud, Switzerland (Commission Cantonale d’éthique de la recherche sur l’être human, No. VD 77/10), Canton Basel, Switzerland (Ethikkommission beider Basel, No. EKBB 242/10), of the Severance Hospital, Yonsei University College of Medicine, South Korea (No. 4-2010-0128) and by the Institutional Bioethics Review Board of Seoul National University Hospital, South Korea (IRB No. H-1004-020-315). Written informed consent was obtained from all study participants adhering to the local ethical guidelines.

### Blood collection and processing

Peripheral blood from all subjects was drawn either up to 30 days before or up to 12 weeks after colonoscopy and prior to any polyp or cancer resection or pre-operative chemotherapy. Blood samples were collected into 4x4 ml BD Vacutainer CPT tubes (Becton Dickinson, Franklin Lakes, NJ). Filled CPT tubes were kept at room temperature and PBMC separation performed within 6 hours according to manufacturer’s instructions. PBMC pellets were resuspended in RNA*later* Solution (Life Technologies, Carlsbad, CA) and stored at -80°C.

### RNA preparation

Automated purification of total RNA was performed on QIAcube by RNeasy Mini kit (Qiagen, Venlo, Netherlands) and included a DNase treatment. RNA concentration was measured by Nanodrop spectrophotometer (Thermo Scientific, Waltham, MA) and RNA integrity was analyzed by Agilent 2100 Bioanalyzer (Agilent Technologies, Santa Clara, CA). Samples with a RIN < 7 were considered of poor quality and discarded. On average RNA showed a RIN of 9±0.5. Isolated total RNA was aliquoted and stored at -80°C. In order to meet the high RNA concentration required by the RT protocol, RNA samples were systematically precipitated following a standard 100% ethanol/3M sodium acetate method.

### Screening design and dataset generation

**Fig 1:**
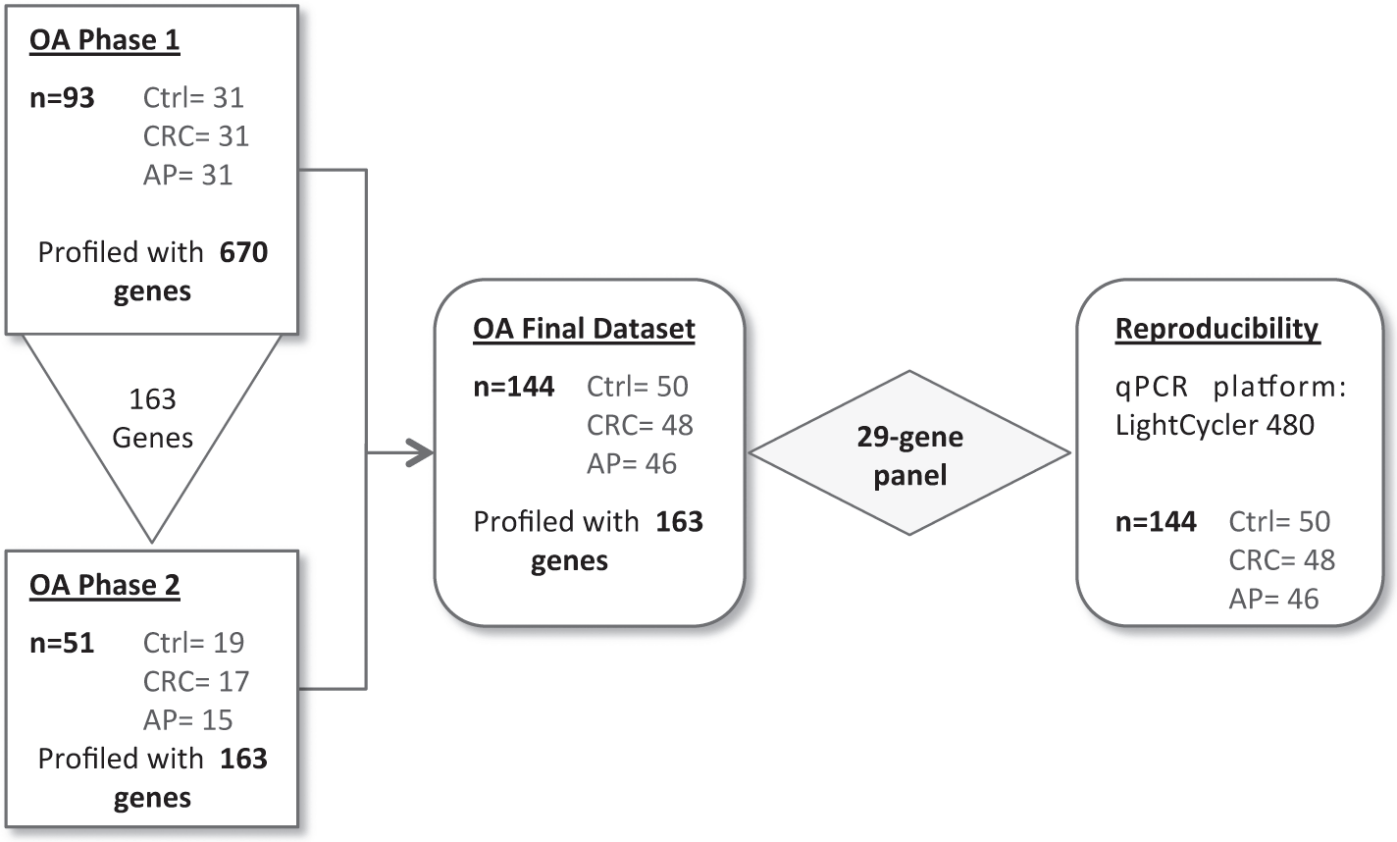
Study design. In a first screening phase performed on the OpenArray system, 670 genes were profiled on 93 samples. Out of these, 163 genes were selected and further tested in phase 2 on additional 51 samples. The final dataset included 144 samples profiled with 163 genes. A 29-gene panel was compiled based on highest power to discriminate AP/CRC from controls by univariate and multivariate analysis. Finally, the 29-gene panel was validated with a LightCycler480 platform, commonly used in clinical laboratories.

In order to find relevant blood biomarkers for CRC, we performed gene expression screening on 144 samples derived from patients with AP or CRC and control subjects. The screening was conceived in 2 phases (Fig 1). First, we profiled 93 samples with a large gene panel. The panel included 667 candidate, of which 42 biomarkers previously identified by our laboratory [8] and 625 new candidate selected from the literature (S1 Table). Three reference genes for qPCR data normalization were also added. The literature search focused on genes, molecular pathways and biological processes considered to be relevant in the tumor-host response such as inflammation and immune response, tumor invasion and metastasis, hematopoiesis, signal transduction pathways (in particular NF-kB pathway), chemokines and cytokines (in particular IL-1, IL-2), extracellular matrix proteins, adhesion molecules and cell surface markers. For each candidate gene, a TaqMan assay was selected from a commercial repository (Life technologies, Carlsbad, CA) (S1 Table) and distributed in three 224-assay Open Array plates, each measuring 12 samples in parallel. Hence, to fully profile 12 samples, three 224-assay plates, thermocycled in parallel, were used. To check for inter-plate variability and reproducibility, the reference gene RPLP0 was assayed for each sample on all 3 plates. RPLP0 standard deviation (SD) analysis of the 3 sample replicates showed a median SD of 0.21 Ct, with an inter-quartile range of 0.14–0.34, indicating that sample measurement was accurate and highly reproducible across the 3-plate series. In this phase we focused on catching the broadest subject biological variability rather than minimizing the technical one, therefore systematic sample replicates were not performed, to allow the profiling of a maximum number of samples.

Out of 670 genes analyzed, 133 showed no expression or a poor PCR amplification. The remaining 534 candidate genes and 3 reference genes were overall well expressed with a median Ct of 22.94 (inter-quartile range: 21.28–25.24) and a median SD of 0.7 (inter-quartile range: 0.59–1.04) (S2 Table). Gene profiles passed through a light filtering step in which they had to satisfy at least one of the following criteria for at least one of the discrimination analysis (*i*.*e*. control versus CRC or control versus AP): a p-value less than 0.1 or a fold-change greater than 1.5 (linear scale). In addition, biomarkers previously identified in our laboratory [8] were retained in this phase regardless their p-value or fold change in order to be assessed in a larger sample set and using a multivariate statistical approach. In total, 160 genes were selected, together with the three reference genes, for the second phase of the screening. This time the 163 genes, measured by 168 assays (5 genes with very low expression had a second assay to validate the measure obtained), were allocated in a single plate (S3 Table). Additional 51 samples were profiled in duplicate with this reduced gene panel to increase the sample size and the statistical power in subsequent analyses. Also, 40 samples already profiled in the phase 1, were re-analyzed to ensure the reproducibility of the measurements across phase 1 and phase 2 and the different plate format (224- *vs*. 168-assay format). The expression levels obtained in both phases for these 40 samples were highly correlated (R^2^ = 0.993) (S1 Fig), prompting us to combine the 93 and 51 samples, profiled for the 163 genes, into a single final dataset (Fig 1). To compile the dataset, the mean values of the 40 samples measured in duplicates were used.

### Nanoliter high throughput qPCR

Nine hundred ng of total RNA was reverse transcribed in 20 μl volume using the high-Capacity cDNA Reverse Transcription kit (Life Technologies, Carlsbad, CA) with random primers, according to the manufacturer’s instructions.

Gene expression profiling was performed using the OpenArray system [22] (Life Technologies, Carlsbad, CA), a nanoliter high throughput real-time PCR platform, allowing 3,072 reactions in a single plate. PCR reactions were performed according to the TaqMan OpenArray real-time plates protocol. Briefly, PCR reaction mixtures containing 2.5 μl GeneAmp Fast PCR Master Mix (Applied Biosystems, Carlsbad, CA), 1 μl TaqMan OpenArray Remix (Applied Biosystems, Carlsbad, CA), 0.3 μl RNase-free water, and 1.2 μl cDNA, were loaded automatically in single or duplicate reaction into the OpenArray plates using an OpenArray AccuFill instrument according to the manufacturer’s protocols. The thermal cycling protocol consisted of 40 cycles at 95°C for 15 seconds and 60°C for 1 minute. At the end of each run, the images, collected before and during the PCR run, were visually inspected to check for sample misloading or plates reading problems. Ct values were computed by the OpenArray analysis software using automatic thresholding with the Ct confidence minimum signal set at 300. Values below the minimum signal setting indicated a failed reaction or no amplification and missing values appeared in the exported data. Most of the time a failed reaction was due to expression levels beyond the limit of detection. Since the maximum Ct detected was inferior to 36, these values were replaced by the arbitrary Ct value of 36. Missing values judged to be caused by technical reasons were replaced by the median Ct values of the concerned gene across all samples. A reference RNA (Xpress Ref Human Universal total RNA) (Qiagen, Venlo, Netherlands) was present as positive control at least once in every PCR run in order to ensure process and reagent stability as well as reproducibility over different PCR runs. Negative controls containing water instead of cDNA were used to rule out possible DNA contamination.

### Real-time qPCR on 384-well plates

Two hundred ng of total RNA was reverse transcribed into cDNA using SuperScript VILO cDNA Synthesis Kit (Invitrogen, Carlsbad, CA) according to manufacturer’s instructions.

RealTime ready Custom RT-qPCR assays (Roche, Basel, Switzerland) based on Universal ProbeLibrary (UPL) technology (S4 Table), were pre-loaded on 384-well plates by the manufacturer. Real-time PCR analysis on 384-well plates was performed on the Lightcycler 480 instrument (Roche, Basel, Switzerland) on 144 samples. PCR reactions were carried out in duplicates in 384-well plates in a total volume of 10 μl. Each well was loaded by an automated pipettor (MICROLAB STARLet, Hamilton Robotics, Reno, NV) with 5 μl of RealTime Ready DNA Probes Master Mix (Roche, Basel, Switzerland) and the cDNA equivalent of 2.5 ng of total RNA. The qPCR program consisted of 2 seconds at 95°C and 30 seconds at 60°C for 40 cycles. Positive and negative controls were generated with each retrotranscription batch and were included in every qPCR run for each target assay. The negative control contained neither RNA nor cDNA to confirm no contamination occurred. The positive control was made with a standardized quantity of Human Universal Reference RNA (Clontech, Mountain View, CA) aliquoted and stored at -80°C. For qPCR run validation, the negative control yielded no amplification or a Crossing point (Cp) value up or equal to 35, and the positive control a Cp value, for each target gene, that fell within a pre-determined range. Cp values were automatically calculated with the LightCycler 480 analysis software according to the 2^nd^ derivative maximum method [23].

### Statistical analysis

PCR data derived from the OpenArray and the LC480 platforms were normalized by the ΔCT method using the mean of three housekeeping genes RPLP0, NACA and TPT1. These genes were selected because they were the most stable in 3 PBMC-related microarray dataset available from the GEO database [24] and also in qPCR analysis performed by us (data not shown).

Gene expression fold change, was defined as ${FC}_{gene}{\;=\;2}^{∆∆Ct}$ with $∆∆Ct\;=\;mean\left({∆Ct}_{Disease}\right)-mean\left({∆Ct}_{Control}\right)$, where ∆Ct is the normalized data.

Wilcoxon rank test [25] was applied to the normalized gene expression data in order to define genes significantly differentially expressed between groups. In addition, in phase 2 screening, Wilcoxon rank test was applied to 500 randomly selected datasets (bootstrap) and significance was set to genes appearing significant (p-value<0.05) in at least 250 bootstraps out of 500.

The multivariate analysis used for feature selection in phase 2 screening included the following methods: K-top scoring pair, a parameter-free, feature selection algorithm [26], and penalized logistic regression method with different algorithms [27, 28].

To evaluate the predictive accuracy of the 29-gene panel, penalized logistic regression models were fitted on the dataset and validated by non-overlapped bootstrap method [29]. Five hundred random datasets were drawn with replacement from dataset; each bootstrap had the same size as the training set. The model was re-fitted at each bootstrap and validated on the out-of-bag samples. The specificity and sensitivity average values over 500 bootstraps were calculated and Receiver Operating Characteristics (ROC) curves were generated by plotting the sensitivity against the false positive rate (1—specificity). Area under the curve (AUC) was calculated.

The Pearson correlation coefficient was used to evaluate linear correlation between genes’ measurements by two instruments.

The R statistics environment was used for statistical analyses.

## Results

### Definition of a 29-gene panel for colorectal cancer and adenoma detection

**Fig 2:**
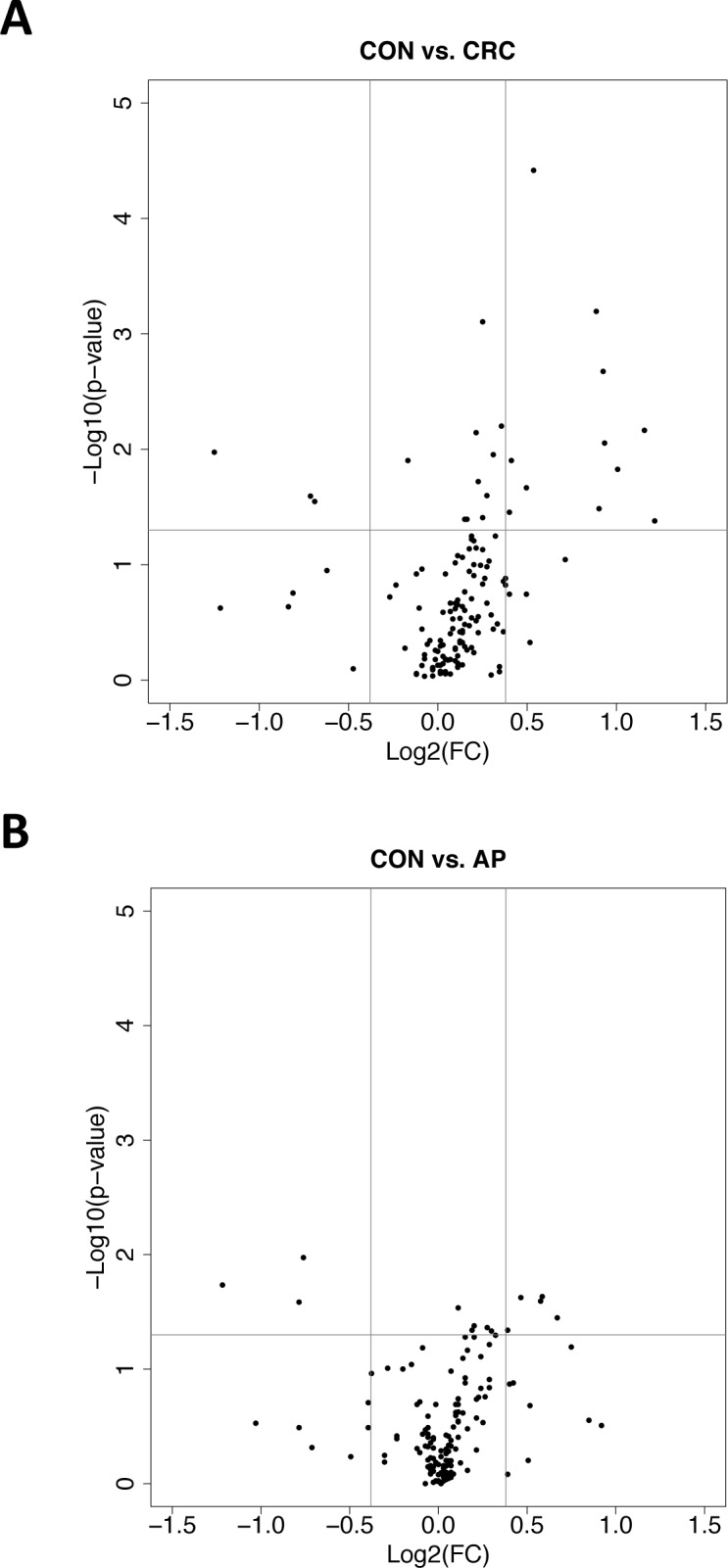
Differential gene expression analysis of 140 genes. The volcano plots summarize the gene expression fold-changes (FC) (*x-axis*) and the p-values (*y-axis*) for the comparisons: **A,** CRC versus control or**, B,** AP versus control. P-value cutoff was fixed at 0.05 (horizontal line) and fold-change threshold at ±0.38 (equivalent to ± 1.3 in linear scale, vertical line). A FC equal to 1 means that the gene is expressed in the group of interest, on average, twice as much than in the control group.

The dataset generated from phase 2 screening (163 genes and 144 samples) was analyzed and filtered for low expression and unstable genes across the two phases, and 20 genes were further discarded, reducing the number of candidate genes to 140. The data were explored in order to define which genes, alone or in combinations, had the highest power to discriminate CRC, AP and AP together with early stage CRC (AP+CRC I-II) from the control group (S3 Table). In addition, the CRC group was compared to AP group, to identify specific genes able to differentiate between CRC and AP. In general, most of the genes appeared to be up-regulated in the CRC and AP groups when compared to the control group. The observed gene expression fold changes were relatively modest, not exceeding a factor of 2.3 (log2 = 1.22) (Fig 2). When a filter based on a FC>1.3 and p-value <0.05 was applied to all group comparisons (CRC/Con, AP/Con, AP+CRCI-II/Con, CRC/AP), we found 28 genes that satisfied both criteria (S3 Table). Among those, 14 discriminate CRC and 8 AP from control group (Fig 2), two of which were common to both conditions (CES1 and IL1B). Seven were specific only for separating AP from CRC and 1 for discriminating AP +CRC I-II. Genes were confirmed to be significant when statistical testing was applied to 500 randomly generated datasets (bootstrap, data not shown).

Multivariate analysis was applied to the dataset to discriminate CRC, AP, AP+CRC from control group. It included KTS-pair [26] and five different algorithms based on penalized logistic regression method [27, 28, 30] for variable selections and model fitting. Genes were ranked according to the frequency of selection by the method used which is summarized by the multivariate score. Thirty-eight genes with a score of at least two were retained to compile the final gene list (S3 Table).

The univariate and multivariate gene lists were then merged, resulting in a final list of 29 genes (Table 2). Interestingly, most of the univariate top-scoring genes appeared to be also the top-scoring genes in the multivariate analysis. Four genes (MAP2K3, MAPK6, CD63, ITGB5), excluded by the filter of the univariate analysis because FC <1.3 but statistically significant (p value<0.05), were “rescued” by the multivariate analysis. Of the other 10 genes not statistically significant and integrated in the final lists thanks to the multivariate approach (GATA2, LTF, MMP9, CXCL10, MSL1, RHOC, FXYD5), the first three showed a FC >1.3.

**Table 2:** 29-gene panel for colorectal cancer and adenoma detection.

Gene Symbol	Con		CRC		AP		CRC/Con		AP+CRCI-II/Con		AP/Con		CRC/AP		Multivariate Score
	mean	SD	mean	SD	mean	SD	p-value	FC	p-value	FC	p-value	FC	p-value	FC	
BCL3*	4.95	0.46	4.41	0.68	4.88	0.61	**3.8E-05**	**1.45**	6.2E-02	1.14	4.7E-01	1.05	**4.5E-04**	**1.39**	12
IL1B*	8.73	0.93	7.85	1.48	8.14	1.10	**6.4E-04**	**1.85**	**8.1E-03**	**1.59**	**2.3E-02**	**1.50**	3.3E-01	1.23	15
PTGS2*	9.65	1.06	8.72	1.59	9.22	1.29	**2.1E-03**	**1.90**	**4.8E-02**	**1.46**	1.3E-01	**1.34**	1.3E-01	**1.42**	8
**MAP2K3**	3.97	0.33	3.61	1.02	3.88	0.49	**6.3E-03**	1.28	1.4E-01	1.16	5.0E-01	1.07	1.0E-01	1.20	3
PTGES*	12.43	2.90	11.27	2.73	12.03	2.26	**6.8E-03**	**2.23**	3.1E-01	**1.52**	8.3E-01	**1.31**	**4.3E-03**	**1.70**	11
PPARG*	12.52	2.27	11.59	2.30	12.58	2.77	**8.8E-03**	**1.91**	1.8E-01	1.20	7.1E-01	-1.04	**3.9E-02**	**1.99**	9
MMP11*	12.53	1.66	13.79	2.77	12.84	2.21	**1.1E-02**	**-2.40**	1.3E-01	**0.56**	6.5E-01	-1.24	**4.3E-02**	**-1.93**	11
CCR1*	5.25	0.65	4.84	0.90	4.98	0.78	**1.2E-02**	**1.33**	**4.2E-02**	1.22	**4.3E-02**	1.21	7.2E-01	1.10	6
EGR1*	6.43	1.50	5.42	1.96	5.91	1.60	**1.5E-02**	**2.01**	**4.6E-02**	**1.72**	2.1E-01	**1.43**	2.4E-01	**1.41**	10
CACNB4*	11.42	0.76	12.14	1.84	11.81	1.78	**2.5E-02**	**-1.64**	1.0E-01	**0.73**	3.2E-01	**-1.31**	3.8E-01	-1.25	15
CES1*	6.65	1.26	7.34	1.79	7.41	1.56	**2.8E-02**	**-1.62**	**2.0E-03**	**0.54**	**1.1E-02**	**-1.69**	9.1E-01	1.05	12
IL8*	7.93	1.58	7.03	1.86	8.03	1.49	**3.3E-02**	**1.87**	6.1E-01	1.20	5.4E-01	-1.07	**7.7E-03**	**2.00**	2
S100A8*	0.48	0.58	0.08	1.03	0.42	0.77	**3.5E-02**	**1.32**	7.3E-01	1.04	9.0E-01	1.04	5.9E-02	1.27	9
CXCL11*	12.00	3.27	12.82	3.42	13.21	3.41	1.8E-01	**-1.75**	**2.0E-02**	**0.44**	**1.8E-02**	**-2.30**	4.4E-01	**1.31**	12
ITGA2*	12.19	2.21	11.85	1.72	11.53	1.45	7.6E-01	1.27	1.1E-01	**1.51**	**3.6E-02**	**1.59**	1.7E-01	-1.25	7
NME1*	10.66	0.96	10.50	1.09	10.27	0.83	5.1E-01	1.11	**2.2E-02**	**1.35**	**4.6E-02**	**1.31**	4.3E-01	-1.18	7
**JUN**	4.77	0.86	5.00	1.20	5.06	0.80	1.5E-01	-1.18	**2.5E-02**	**0.77**	9.8E-02	-1.22	8.7E-01	1.04	5
**TNFSF13B**	4.35	0.52	4.11	0.83	4.51	0.54	1.5E-01	1.19	3.8E-01	0.96	9.1E-02	-1.11	**5.6E-03**	**1.32**	1
**CXCR3**	9.04	0.82	9.32	1.00	8.80	0.71	1.9E-01	-1.21	6.8E-01	1.02	7.8E-02	1.18	**6.9E-03**	**-1.43**	3
**MAPK6**	6.99	0.28	6.74	0.46	7.03	0.37	**7.9E-04**	1.19	4.5E-01	1.02	8.2E-01	-1.03	**5.6E-04**	1.22	6
**CD63**	3.05	0.31	2.82	0.62	3.04	0.56	**1.9E-02**	1.17	4.1E-01	1.03	5.8E-01	1.01	1.2E-01	1.16	2
**ITGB5**	6.16	0.98	6.00	1.16	5.84	0.95	5.5E-01	1.12	2.2E-01	1.17	5.1E-02	1.25	2.2E-01	-1.12	6
**GATA2**	7.96	1.06	8.14	1.08	8.36	0.90	5.3E-01	-1.13	1.8E-01	0.78	2.0E-01	**-1.31**	6.0E-01	1.16	6
**LTF**	14.08	3.64	13.97	4.35	13.17	3.49	6.2E-01	1.08	6.9E-01	1.23	3.1E-01	**1.89**	6.2E-01	**-1.74**	8
**MMP9**	14.11	3.38	13.59	3.77	13.61	3.41	4.7E-01	**1.43**	8.0E-01	1.21	6.3E-01	**1.42**	8.4E-01	1.01	7
**CXCL10**	8.47	0.67	8.43	1.16	8.55	0.93	8.5E-01	1.03	4.1E-01	0.96	3.4E-01	-1.06	6.2E-01	1.08	6
**MSL1**	4.88	0.39	4.87	0.67	4.91	0.46	8.8E-01	1.01	9.2E-01	0.99	6.0E-01	-1.02	4.1E-01	1.03	4
**RHOC**	5.34	0.42	5.45	0.80	5.28	0.52	2.4E-01	-1.08	9.6E-01	0.98	4.6E-01	1.04	1.1E-01	-1.13	4
**FXYD5**	1.21	0.44	1.25	0.64	1.32	0.44	4.5E-01	-1.03	4.8E-01	0.94	1.9E-01	-1.08	6.3E-01	1.04	3

The analysis has been carried out with the delta Ct values obtained on 144 samples. P-values were determined by the Wilcoxon rank sum test. Fold-change (FC) inductions are expressed in linear values for a more intuitive reading. FC >1.3 and p-values <0.05 are in bold. Genes marked with * were the 15 top-ranked genes by univariate analysis for AP and CRC discrimination. The multivariate score represents the frequency by which each gene appeared during the analysis and is calculated by summing the gene presence in all combinations/fitted models for all group analyses. The score could range from 0 (no selection) to 18 (presence in every model/combination) and genes with a score of at least two were retained to compile the final list.

Functional analysis conducted with the Ingenuity Pathway Analysis package (IPA; www.qiagen.com/ingenuity), revealed that the panel was enriched in genes involved in leukocytes migration and chemotaxis (CCR1, CXCL10, CXCL11, CXCR3, IL1B, IL8, ITGA2, MMP9, S100A8) (Table 3). These cellular functions are tightly related to immune cell trafficking and inflammation. Highly represented were also genes involved in cell proliferation and differentiation (BCL3, CD63, EGR1, GATA2, JUN, LTF, MAPK6, MMP11, NME1, PPARG, TNFSF13B), reflecting a possible role in hematopoiesis.

**Table 3:** Functional analysis of the 29-gene panel.

IPA Functional Category	p-Value	Genes
Leukocytes migration and chemotaxis	2.51E-14	18
Hematological system development	6.74E-11	14
Gene expression	3.78E-11	15
Cell death and survival	1.09E-09	17
Cell signaling and interaction	1.47E-09	15
Cellular growth and proliferation	4.02E-09	21

The table reports the most significantly represented biological functions within the gene panel. P-values measure the likelihood that the association between a set of biomarkers and a given Ingenuity Pathway Analysis (IPA) functional category is random. The p-value is calculated using the right-tailed Fisher Exact Test. The number of genes associated with a specific function is reported in the last column.

### Validation of the 29-gene panel

**Fig 3:**
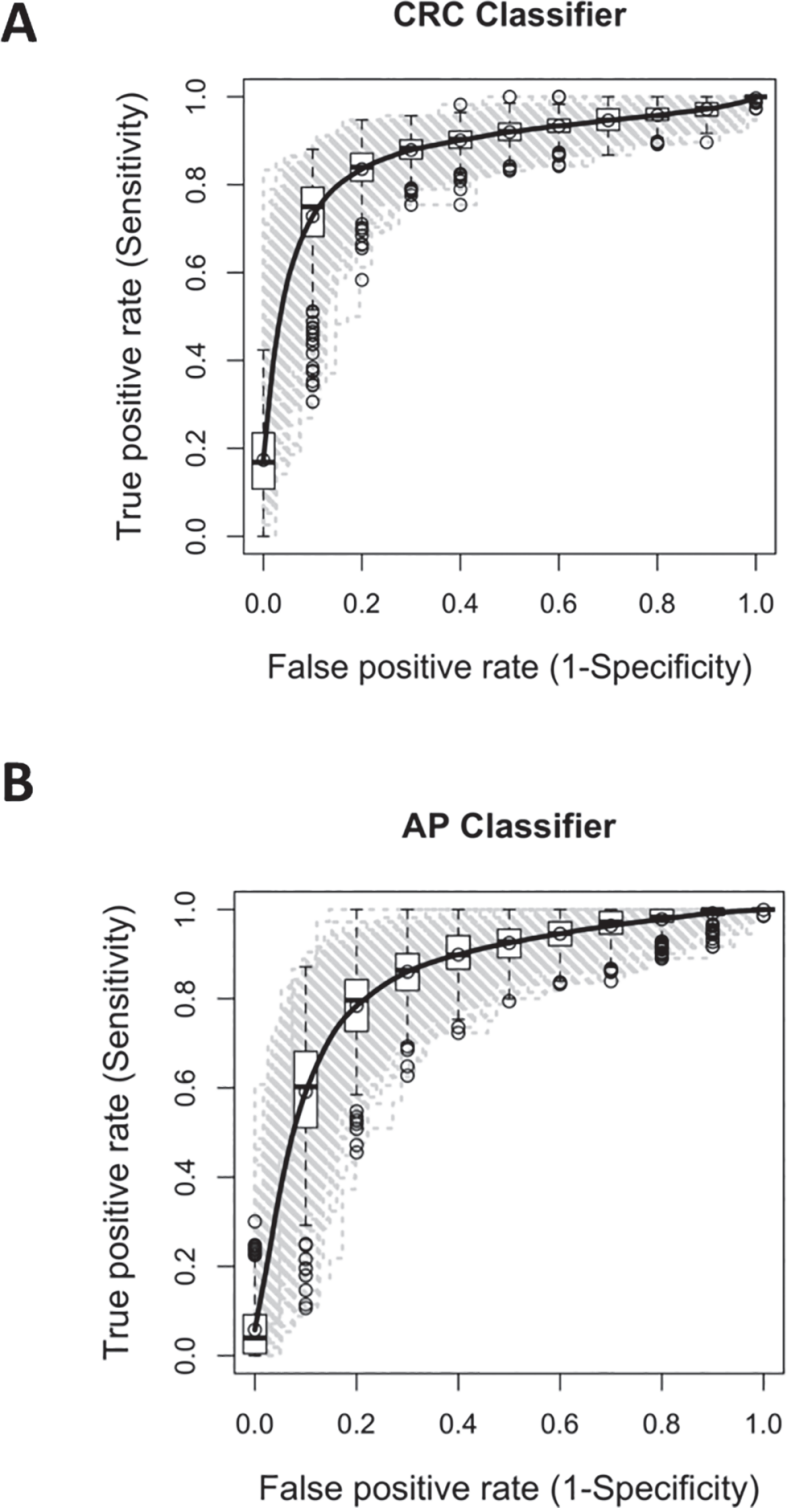
Receiving Operating Characteristics (ROC) analysis. **A.** Summary of the false and true positive rates of the 29-gene panel in classifying CRC cases. **B.** Summary of the false and true positive rates of the 29-gene panel in classifying AP cases. Analyses were performed using 500 bootstrap validations. The boxplots represent the distribution of the 500 bootstraps. The black line represents the average values over 500 bootstraps for clinical specificity and sensitivity.

To evaluate the clinical relevance of our 29-gene panel, in particular its predictive accuracy, penalized logistic regression was applied to the dataset and fitted models were validated by non-overlapped bootstrap method. Models could discriminate CRC or AP >1cm from controls with an average sensitivity of 75% and 59%, respectively. The average specificity, defined as the number of controls correctly classified over the total number of controls, was 91%. ROC analysis determined an AUC of 0.88 (0.83–0.92, 95% CI) and of 0.85 (0.78–0.91, 95%CI) for CRC or AP detection, respectively (Fig 3). When the same approach was applied to the 15 top-ranked genes for CRC or AP discrimination by univariate analysis only (Table 2), predictive accuracy drastically decreased. When specificity was set at 91%, CRC were detected with a sensitivity of 65% and AP with a sensitivity of 37%, with an AUC of 0.86 (0.77–0.84, 95% CI) and 0.77 (0.70–0.82, 95% CI), respectively. This result supported our choice of integrating univariate and multivariate approach for gene selection: genes that otherwise would have been discarded because not meeting the fixed p-value and FC criteria, were indeed valuable for CRC and AP detection as deemed by multivariate methods.

### Assay migration to a 384-well plate qPCR platform

The OpenArray platform demonstrated to be valuable for high throughput gene expression profiling and biomarker discovery. However, this platform is not suitable in a routine clinical laboratory setting, in which easiness of use, flexibility and low costs are preferred. With the aim of developing the 29-gene panel into a widely used CRC screening test, we evaluated the panel behavior on a qPCR platform that is commonly adopted by clinical laboratories. The 144 samples were profiled with the 29-gene panel using a LightCycler 480 (LC480) instrument and a set of commercially available probe-based assays (Universal ProbeLibrary, Roche), preloaded on 384-well plates.

**Fig 4:**
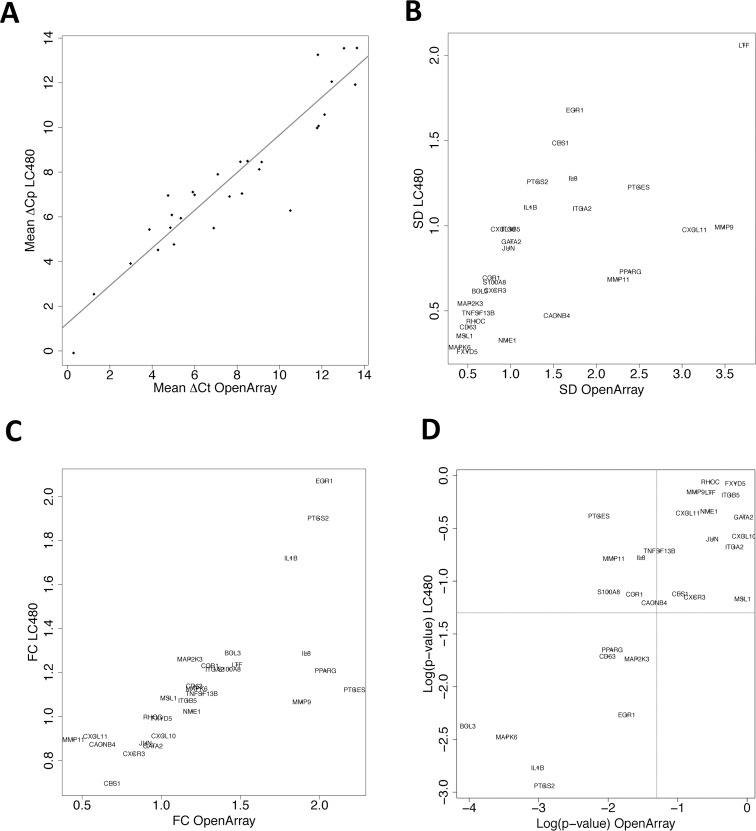
Validation of the 29-gene panel on the LightCycler480 qPCR platform. Scatter plots comparing analyses performed for each gene on the datasets generated on the LC480 and OpenArray platforms. The following variables have been used: **A.** Mean normalized expression values (ΔCp and ΔCt) (R^2^: 0.933), **B.** Mean standard deviations (SD) relative to each target gene measured, **C.** Gene expression fold changes between the CRC and the control group (linear absolute values), **D.** p-values from statistical testing between the CRC and the control group (log transformed). Lines represent a p-value<0.05. Gene names have been overlapped to the graphs.

Correlation and linear regression analysis showed that gene expression levels measured on the two platforms were highly comparable (correlation coefficient: 0.933) (Fig 4A). In general, gene expression showed similar variance across the samples (Fig 4B). However, a group of lowly expressed genes displayed measurements with smaller standard deviations on the LC480 platform than on the OpenArray one, suggesting that the assays on the LC480 instrument are more accurate. When we repeated the differential gene expression analysis between controls and CRC groups with the LC480 dataset, we found that relative abundance and statistical significance for the 29 genes were similar or with the similar trend to what observed on the OpenArray dataset (Fig 4C and 4D). However, for five genes (PTGES, MMP11, IL8, CCR1, S100A8) statistical significance was lost when measured on the LC480.

## Discussion and Conclusions

In this work we have identified a 29-gene panel expressed in PBMC, capable of discriminating individuals with AP >1cm or CRC from healthy individuals. Penalized logistic regression analysis correctly classified 75%, 59% (sensitivity) and 91% (specificity) of CRC, AP and controls, respectively.

The approach we used is different compared to existing screening tests for CRC, as it is based on PBMC genes expression profiles. This leverages the well-established concept of tumor-host interaction and contribution of bone marrow-derived cells to tumor progression [16, 17]. It is of interest the fact that AP, considered premalignant lesions, are also detected, albeit with a lower sensitivity, by the 29-gene panel. Indeed, inflammation is associated with neoplastic colonic polyp formation [31] and inflammatory bowel diseases, in particular colitis ulcerosa, are a risk factor for CRC [32]. Importantly, non-steroidal anti-inflammatory drugs protect against CRC development [33], prevent adenoma formation in experimental models of familial adenomatous polyposis coli [34] and reverse gene expression changes in the normal colon to adenoma sequence [35]. This reinforces the notion that the proposed approach might be developed for AP and early CRC detection as more effective alternative to fecal occult blood-based tests. The pool of 670 candidate genes used for the screening included many host genes and pathways involved in inflammation, immune response and tumor progression. Importantly, these genes were not chosen based on a preceding genome-wide screen, but based on existing knowledge (i.e. literature and own data) and hypotheses (i.e. role of inflammation in cancer progression). The majority of these 29 genes of the panel are mediators/regulators of inflammation, cell motility, cell survival, cell signaling and proliferation (Table 2 and 3). This is consistent with the notion that tumor-mobilized bone marrow-derived circulating myelomoncytic cells are in a state of activation in response to tumor-released factors. We have previously shown that tumor-released PlGF and KitL are capable of modulating the differentiation program of CD11b^+^ cells mobilized from the bone marrow in response to the growing primary tumor, thereby generating pro-angiogenic or pro-metastatic CD11b^+^ cells, respectively [36, 37].

We recognize that genes associated to inflammatory processes might be similarly modulated in inflammatory conditions/diseases other than CRC or AP, possibly leading to reduced signature specificity. However, the weight of possibly “non-specific” genes is moderated by the information carried by other genes in the panel. We are planning to evaluate the gene panel in an independent test set of samples collected during this clinical study, including several inflammatory conditions, in particular inflammatory bowel diseases and to use this sample set to optimize a predictive algorithm highly specific for CRC and AP detection.

Technically, our discovery strategy leveraged the recent development of accurate and sensitive high-throughput qPCR platforms based on microfluidic technologies, such as the OpenArray [22]. The apparent disadvantage of this approach, the screening of only a limited part of the transcriptome, is overcome by several advantages. In particular, the high analytical sensitivity typical of qPCR, reached with relatively low amounts of native, non-amplified RNA, together with a direct transcript measure, will make further analytical validation steps unnecessary. In addition, the rapidity of sample processing due to the high throughput technology, with the analysis of more than hundred samples per day, and the easiness of data mining and bioinformatic analysis, greatly balanced the advantages of a genome-wide approach, like microarray and RNA-seq. With the aim of translating our gene panel into an assay easily implementable as a routine lab test, we performed a technology transfer from the high throughput platform to a standard 384-well plate qPCR platform using plates preloaded with specific target gene assays. As shown, the gene expression profiles and differential analysis results obtained from the two platforms were comparable. We therefore concluded that the LC480 platform is suitable to implement a CRC screening assay based on the 29-gene panel. Nevertheless, it would be interesting to reanalyze the samples with a genome wide approach (e.g. RNAseq) and to compare the discriminatory power of the newly identified genes to the 29-gene panel described here. Additional genes issued from such an approach might be eventually added to the current signature to improve sensitivity and specificity of the test.

The findings reported here are in line with the recently reported results obtained from a pilot monocentric study in which the feasibility of the use of a PBMC-derived signature to detect CRC and AP was demonstrated [8]. The significance of the study was limited, however, by the small sample size and by the low number of CRC samples compared to AP. The present study was designed to independently validate this pilot study in a multicenter, case-control study. The two studies also differed from a technical point of view as numerous changes were introduced in the assay procedures, in particular blood collection, PBMC isolation and the qPCR chemistry, which made necessary a fully new development and assessment.

In spite of the major clinical and technical differences in these studies, 8 genes were found common to the 29-gene panel reported here and the 42-gene panel previously reported [8]. Four of those, would have been excluded from the 29-gene panel without the decision of pushing to the phase 2 of the screening, genes identified by Nichita *et al*. but not significant in phase 1. Moreover, the predictive accuracy reported in the two studies is very similar, thereby demonstrating the consistency and robustness of our approach. The fact that the two panels overlap only for a fraction of genes could be explained by the fact that the biomarker discovery reported by Nichita *et al*. focused predominantly on adenoma samples rather than CRC. However, it is already known that different gene signatures may carry the same biological information as it was reported in gene-expression signatures in breast cancer [38].

In conclusion, we have discovered and characterized a 29-gene panel in PBMC for the detection of colorectal adenomas and CRC. The signature can discriminate AP >1cm and CRC from controls with an average sensitivity of 59% and 75%, respectively, and a specificity of 91%. We also demonstrated that the gene panel could be easily transferred and implemented into a medical laboratory-friendly assay, which is a key step in the development of a new cost-effective, simple blood-based colorectal cancer screening test. The identified signature will be the basis for developing a decisional algorithm that will be validated for its prospective discriminatory value on the remaining samples collected in this case-control multicenter study. The availability of a larger number of AP>1 cm and early stage CRC samples, as well as the presence of patients with inflammatory diseases or other type of tumors, will allow to finely tune the algorithm to be highly sensitive and specific to precancerous and early stage cancerous lesion.

## Supporting Information

S1 Fig Linear regression between mean Ct values obtained from the two screening phases. Forty samples were analyzed in both phase 1 and phase 2 and Ct values compared. Ct1 refers to Ct values obtained in the phase 1 and Ct2 in the phase 2. Mean Ct values obtained in the two phases were highly correlated (R^2^ = 0.993).
(TIFF)

S1 Table Annotated list with the 670 genes tested in the screening. For each of the gene is reported the official gene symbol, the full gene name, the RNA reference sequence database ID (RefSeq), the TaqMan assay ID (Life Technologies, Carlsbad, CA) used during the screening as well as, if any, the alternative assay used in phase 2 (TaqMan assay 2), and at last the genes selected for phase 2 screening.
(XLSX)

S2 Table Summary of the univariate analysis performed during phase 1 screening. In this phase 93 PBMC samples, equally distributed among controls (Con), adenomatous polyps >1cm (AP) and CRCs stage I-IV (stage I-II, n = 17), were profiled with 670 genes. The table displays only those 534 genes showing expression levels above the limit of detection for more than 50% of the samples or a good PCR amplification. Mean, standard deviation (SD) and median values are reported for four main analysis groups (Con, AP, CRC and AP plus CRC (AP-CRC)) and for two subgroups: CRC stages I-II only (CRCI-II) and AP plus CRCI-II (AP-CRCI-II). During the univariate analysis, the disease groups/subgroups were compared to the control one and the AP group was evaluated against the CRC one. For each gene and for each group analysis, p-values derived from Student’s t-test and Wilcoxon rank sum test and fold-change (FC) linear values, derived from the mean or median, are reported. In addition, the table indicates if the gene was selected for the phase 2 screening (163 genes) and in the final 29-gene panel.
(XLSX)

S3 Table Summary of the univariate and multivariate analysis performed during phase 2 screening. In this phase 144 PBMCs samples, including 50 controls (Con), 46 adenomatous polyps >1cm (AP) and 48 CRCs stage I-IV (stage I-II, n = 24), were profiled with 163 genes. Univariate and multivariate analysis were conducted only on the 140 genes showing stable and reproducible measurements between the two phases. For the univariate analysis, the same settings explained in S2 Table were applied, and the results were reported similarly. Multivariate analysis was applied only for the discrimination of the main groups (CRC, AP, AP-CRC) from control one. It included KTS-pair and five different penalized logistic regression algorithms for variable selections and model fitting. Selection of a given gene by one multivariate method, for one particular group analysis, is indicated by 1, whereas non-selection is indicated by 0. The frequency by which each gene appeared during the analysis is summarized by the Multivariate Score, calculated by summing the gene presence in all combinations/fitted models for all group analyses. The score could range from 0 to 18 and genes with a score of at least two were retained to compile the final gene list. (nd: not determined, NA: not applicable).
(XLSX)

S4 Table Final 29-gene panel. RealTime ready Custom RT-qPCR assays (Roche, Basel, Switzerland) used to validate the gene panel on the LightCycler 480 instrument are reported, including forward and reverse primer sequences and the associated UPL probe ID. The assays were pre-loaded on 384-well plates. Reference genes used for PCR values normalization are marked with *.
(PDF)

## References

[1] Jemal A, Siegel R, Xu J, Ward E. Cancer statistics, 2010. CA Cancer J Clin. 2010;60: 277–300. 10.3322/caac.20073 20610543

[2] Levin B, Lieberman DA, McFarland B, Andrews KS, Brooks D, Bond J, et al Screening and surveillance for the early detection of colorectal cancer and adenomatous polyps, 2008: a joint guideline from the American Cancer Society, the US Multi-Society Task Force on Colorectal Cancer, and the American College of Radiology. Gastroenterology. 2008;134: 1570–1595. 10.1053/j.gastro.2008.02.002 18384785

[3] von Karsa L, Patnick J, Segnan N, Atkin W, Halloran S, Lansdorp-Vogelaar I, et al European guidelines for quality assurance in colorectal cancer screening and diagnosis: overview and introduction to the full supplement publication. Endoscopy. 2013;45: 51–59. 10.1055/s-0032-1325997 23212726 PMC4482205

[4] Parente F, Boemo C, Ardizzoia A, Costa M, Carzaniga P, Ilardo A, et al Outcomes and cost evaluation of the first two rounds of a colorectal cancer screening program based on immunochemical fecal occult blood test in northern Italy. Endoscopy. 2013;45: 27–34. 10.1055/s-0032-1325800 23254404

[5] Jean-Jacques M, Kaleba EO, Gatta JL, Gracia G, Ryan ER, Choucair BN. Program to improve colorectal cancer screening in a low-income, racially diverse population: a randomized controlled trial. Ann Fam Med. 2012;10: 412–417. 10.1370/afm.1381 22966104 PMC3438208

[6] von Wagner C, Baio G, Raine R, Snowball J, Morris S, Atkin W, et al Inequalities in participation in an organized national colorectal cancer screening programme: results from the first 2.6 million invitations in England. Int J Epidemiol. 2011;40: 712–718. 10.1093/ije/dyr008 21330344

[7] Han M, Liew CT, Zhang HW, Chao S, Zheng R, Yip KT, et al Novel blood-based, five-gene biomarker set for the detection of colorectal cancer. Clin Cancer Res. 2008;14: 455–460. 10.1158/1078-0432.CCR-07-1801 18203981

[8] Nichita C, Ciarloni L, Monnier-Benoit S, Hosseinian S, Dorta G, Ruegg C. A novel gene expression signature in peripheral blood mononuclear cells for early detection of colorectal cancer. Aliment Pharmacol Ther. 2014;39: 507–517. 10.1111/apt.12618 24428642

[9] Marshall KW, Mohr S, Khettabi FE, Nossova N, Chao S, Bao W, et al A blood-based biomarker panel for stratifying current risk for colorectal cancer. Int J Cancer. 2010;126: 1177–1186. 10.1002/ijc.24910 19795455

[10] Honda M, Sakai Y, Yamashita T, Sakai A, Mizukoshi E, Nakamoto Y, et al Differential gene expression profiling in blood from patients with digestive system cancers. Biochem Biophys Res Commun. 2010;400: 7–15. 10.1016/j.bbrc.2010.07.123 20682290

[11] Sharma P, Sahni NS, Tibshirani R, Skaane P, Urdal P, Berghagen H, et al Early detection of breast cancer based on gene-expression patterns in peripheral blood cells. Breast Cancer Res. 2005;7: R634–644. 16168108 10.1186/bcr1203 PMC1242124

[12] Twine NC, Stover JA, Marshall B, Dukart G, Hidalgo M, Stadler W, et al Disease-associated expression profiles in peripheral blood mononuclear cells from patients with advanced renal cell carcinoma. Cancer Res. 2003;63: 6069–6075. 14522937

[13] Burczynski ME, Twine NC, Dukart G, Marshall B, Hidalgo M, Stadler WM, et al Transcriptional profiles in peripheral blood mononuclear cells prognostic of clinical outcomes in patients with advanced renal cell carcinoma. Clin Cancer Res. 2005;11: 1181–1189. 15709187

[14] Showe MK, Vachani A, Kossenkov AV, Yousef M, Nichols C, Nikonova EV, et al Gene expression profiles in peripheral blood mononuclear cells can distinguish patients with non-small cell lung cancer from patients with nonmalignant lung disease. Cancer Res. 2009;69: 9202–9210. 10.1158/0008-5472.CAN-09-1378 19951989 PMC2798582

[15] Osman I, Bajorin DF, Sun TT, Zhong H, Douglas D, Scattergood J, et al Novel blood biomarkers of human urinary bladder cancer. Clin Cancer Res. 2006;12: 3374–3380. 16740760 10.1158/1078-0432.CCR-05-2081

[16] Lorusso G, Ruegg C. The tumor microenvironment and its contribution to tumor evolution toward metastasis. Histochem Cell Biol. 2008;130: 1091–1103. 10.1007/s00418-008-0530-8 18987874

[17] Coussens LM, Werb Z. Inflammation and cancer. Nature. 2002;420: 860–867. 12490959 10.1038/nature01322 PMC2803035

[18] Quackenbush J. Microarray analysis and tumor classification. N Engl J Med. 2006;354: 2463–2472. 16760446 10.1056/NEJMra042342

[19] Xuan J, Yu Y, Qing T, Guo L, Shi L. Next-generation sequencing in the clinic: promises and challenges. Cancer Lett. 2013;340: 284–295. 10.1016/j.canlet.2012.11.025 23174106 PMC5739311

[20] Bernard PS, Wittwer CT. Real-time PCR technology for cancer diagnostics. Clin Chem. 2002;48: 1178–1185. 12142370

[21] Espinosa E, Redondo A, Vara JA, Zamora P, Casado E, Cejas P, et al High-throughput techniques in breast cancer: a clinical perspective. Eur J Cancer. 2006;42: 598–607. 16431104 10.1016/j.ejca.2005.11.021

[22] Henriquez-Hernandez LA, Valenciano A, Herrera-Ramos E, Lloret M, Riveros-Perez A, Lara PC. High-throughput genotyping system as a robust and useful tool in oncology: experience from a single institution. Biologicals. 2013;41: 424–429. 10.1016/j.biologicals.2013.09.006 24103542

[23] Rasmussen R (2001) Quantification on the LightCycler In: Meyer S, Wittwer C, Nakagawara K, editors. Rapid Cycle Real-time PCR, Methods and Applications. Heidelberg: Springer Press pp. 21–34.

[24] Popovici V, Goldstein DR, Antonov J, Jaggi R, Delorenzi M, Wirapati P. Selecting control genes for RT-QPCR using public microarray data. BMC Bioinformatics. 2009;10: 42 10.1186/1471-2105-10-42 19187545 PMC2640357

[25] Wilcoxon F. Individual comparisons of grouped data by ranking methods. J Econ Entomol. 1946;39: 269 20983181 10.1093/jee/39.2.269

[26] Tan AC, Naiman DQ, Xu L, Winslow RL, Geman D. Simple decision rules for classifying human cancers from gene expression profiles. Bioinformatics. 2005;21: 3896–3904. 16105897 10.1093/bioinformatics/bti631 PMC1987374

[27] Park MY, Hastie T. L1 regularization path algorithm for generalized linear models. 2007;69: 659–677.

[28] Friedman J, Hastie T, Tibshirani R. Regularization Paths for Generalized Linear Models via Coordinate Descent. J Stat Softw. 2010;33: 1–22. 20808728 PMC2929880

[29] Steyerberg EW, Harrell FE Jr., Borsboom GJ, Eijkemans MJ, Vergouwe Y, Habbema JD. Internal validation of predictive models: efficiency of some procedures for logistic regression analysis. J Clin Epidemiol. 2001;54: 774–781. 11470385 10.1016/s0895-4356(01)00341-9

[30] Goeman JJ. L1 penalized estimation in the Cox proportional hazards model. Biom J. 2010;52: 70–84. 10.1002/bimj.200900028 19937997

[31] Bilinski C, Burleson J, Forouhar F. Inflammation associated with neoplastic colonic polyps. Ann Clin Lab Sci. 2012;42: 266–270. 22964614

[32] Grivennikov SI. Inflammation and colorectal cancer: colitis-associated neoplasia. Semin Immunopathol. 2013;35: 229–244. 10.1007/s00281-012-0352-6 23161445 PMC3568220

[33] Brown JR, DuBois RN. COX-2: a molecular target for colorectal cancer prevention. J Clin Oncol. 2005;23: 2840–2855. 15837998 10.1200/JCO.2005.09.051

[34] Oshima M, Murai N, Kargman S, Arguello M, Luk P, Kwong E, et al Chemoprevention of intestinal polyposis in the Apcdelta716 mouse by rofecoxib, a specific cyclooxygenase-2 inhibitor. Cancer Res. 2001;61: 1733–1740. 11245490

[35] Galamb O, Spisak S, Sipos F, Toth K, Solymosi N, Wichmann B, et al Reversal of gene expression changes in the colorectal normal-adenoma pathway by NS398 selective COX2 inhibitor. Br J Cancer. 2010;102: 765–773. 10.1038/sj.bjc.6605515 20087348 PMC2837560

[36] Laurent J, Hull EF, Touvrey C, Kuonen F, Lan Q, Lorusso G, et al Proangiogenic factor PlGF programs CD11b(+) myelomonocytes in breast cancer during differentiation of their hematopoietic progenitors. Cancer Res. 2011;71: 3781–3791. 10.1158/0008-5472.CAN-10-3684 21507936

[37] Kuonen F, Laurent J, Secondini C, Lorusso G, Stehle JC, Rausch T, et al Inhibition of the Kit ligand/c-Kit axis attenuates metastasis in a mouse model mimicking local breast cancer relapse after radiotherapy. Clin Cancer Res. 2012;18: 4365–4374. 22711708 10.1158/1078-0432.CCR-11-3028

[38] Sotiriou C, Pusztai L. Gene-expression signatures in breast cancer. N Engl J Med. 2009;360: 790–800. 10.1056/NEJMra0801289 19228622

