# Supplementary figures and images for: Discovery of a 29-Gene Panel in Peripheral Blood Mononuclear Cells for the Detection of Colorectal Cancer and Adenomas Using High Throughput Real-Time PCR
Source: PLoS One. 2015 Apr 13;10(4):e0123904. doi: 10.1371/journal.pone.0123904 (PMC4395254; doi:10.1371/journal.pone.0123904)

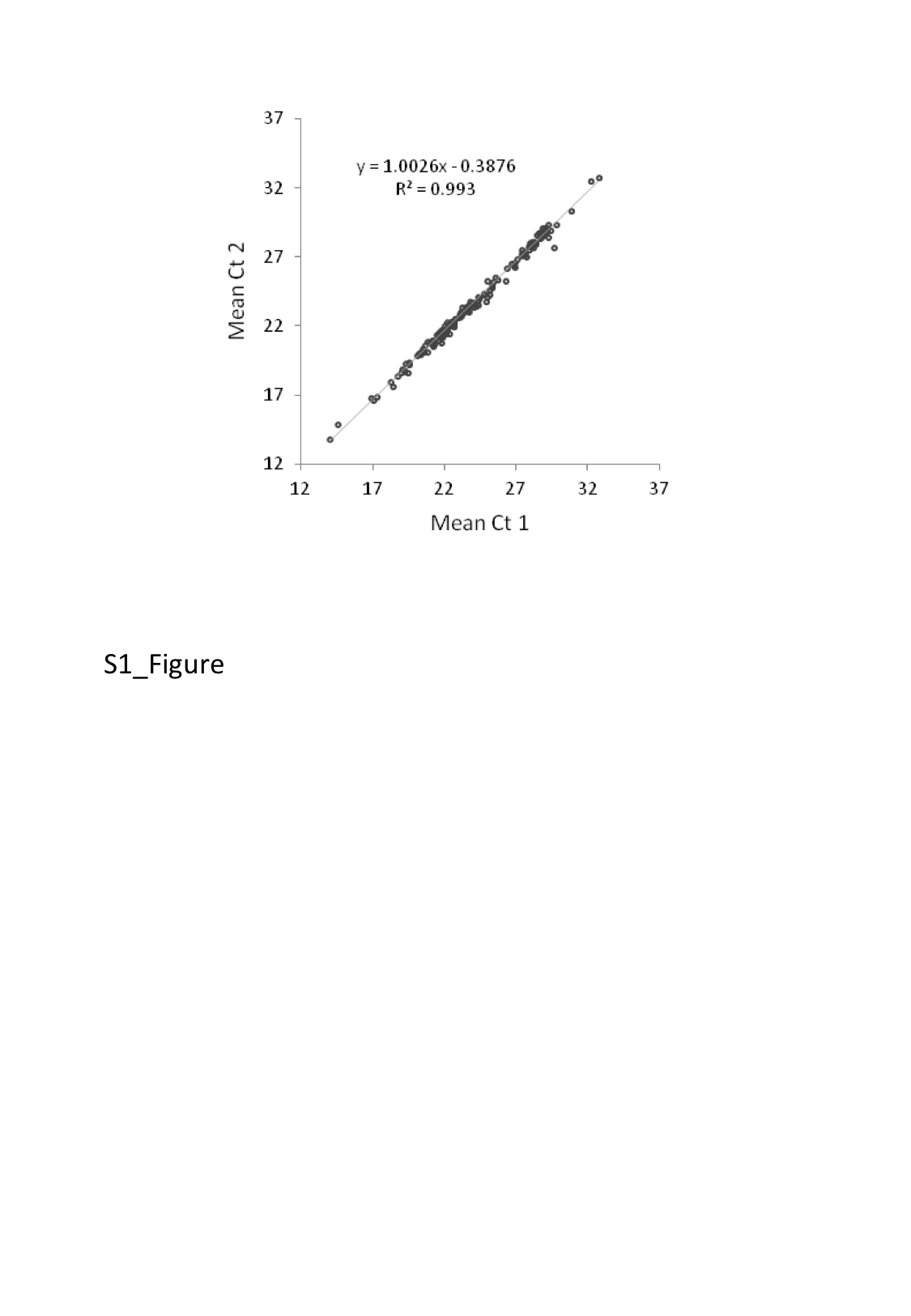

Supplement: S1 Fig — Forty samples were analyzed in both phase 1 and phase 2 and Ct values compared. Ct1 refers to Ct values obtained in the phase 1 and Ct2 in the phase 2. Mean Ct values obtained in the two phases were highly correlated (R2 = 0.993). (TIFF) [file pone.0123904.s001.tiff]
