# Supplementary material for: Discovery of a 29-Gene Panel in Peripheral Blood Mononuclear Cells for the Detection of Colorectal Cancer and Adenomas Using High Throughput Real-Time PCR
Source: PLoS One. 2015 Apr 13;10(4):e0123904. doi: 10.1371/journal.pone.0123904 (PMC4395254; doi:10.1371/journal.pone.0123904)
Supplement: S4 Table — RealTime ready Custom RT-qPCR assays (Roche, Basel, Switzerland) used to validate the gene panel on the LightCycler 480 instrument are reported, including forward and reverse primer sequences and the associated UPL probe ID. The assays were pre-loaded on 384-well plates. Reference genes used for PCR values normalization are marked with *. (PDF) [file pone.0123904.s005.pdf]

S2 Table

RefSeq	Gene Symbol	Gene Description	Forward Primer Sequence	Reverse Primer Sequence	UPL Probe ID
NM_005178	BCL3	B-cell CLL/lymphoma 3	ACAACAACCTACGGCAGACA	CCACAGACGGTAATGTGGTG	76
NM_001005746	CACNB4	calcium channel, voltage-dependent, beta 4 subunit	TCCAAGCACAGCTATCTCCTT	CCCTCTTTCACCAGCCTTC	138
NM_001295	CCR1	chemokine (C-C motif) receptor 1	AGTGATTTCCACAGTGACTCCA	GGCAGATGCTGGCTACTGAT	95
NM_001040034	CD63	CD63 molecule	GAATGAAATGTGTGAAGTTCTTGC	GCAATCAGTCCCACTGCAC	18
NM_001025194	CES1	carboxylesterase 1 (monocyte/macrophage serine esterase 1)	CAGGAGTTTGGCTGGTTGAT	CAGTTGCCCTTCGGAGAGT	136
NM_001565	CXCL10	chemokine (C-X-C motif) ligand 10	AAAAGGTATGCAATCAAATCTGC	AAGAATTTGGGCCCCTTG	86
NM_005409	CXCL11	chemokine (C-X-C motif) ligand 11	TTGTGTGCTACAGTTGTTCAAGG	TCTGCCACTTTCACTGCTTTTA	81
NM_001142797	CXCR3	chemokine (C-X-C motif) receptor 3	ACCACAAGCACCAAAGCAG	GGCGTCATTTAGCACTTGGT	27
NM_001964	EGR1	early growth response 1	AGCACCTGACCGCAGAGT	GGCAGTCGAGTGGTTTGG	54
NM_144779	FXYD5	FXYD domain containing ion transport regulator 5	ACCACGTCCAGTTCTTCAGC	GGGCTGGAGTTCTGTGTAGACT	45
NM_032638	GATA2	GATA binding protein 2	CACAAGATGAATGGGCAGAA	TGACAATTTGCACAACAGGTG	117
NM_000576	IL1B	interleukin 1, beta	AGCTGATGGCCCTAAACAGA	TCGGAGATTCGTAGCTGGAT	85
NM_000584	IL8	interleukin 8	TAGCCAGGATCCACAAGTCC	CTGTGAGGTAAGATGGTGGCTA	98
NM_002203	ITGA2	integrin, alpha 2 (CD49B, alpha 2 subunit of VLA-2 receptor)	AACATGAGCCTCGGCTTG	GCCCACAGAGGACCACAT	154
NM_002213	ITGB5	integrin, beta 5	GCATGCAGCACCAAGAGAG	GCAGGTCTGGTTGTCAGGTT	40
NM_002228	JUN	jun proto-oncogene	AGTCAGGCAGACAGACAGACAC	AAAATAAGATTTGCAGTTCGGACTAT	20
NM_001199149	LTF	lactotransferrin	TAAGGTGGAACGCCTGAAAC	CCATTTCTCCCAAATTTAGCC	22
NM_145109	MAP2K3	mitogen-activated protein kinase kinase 3	CGAGTTTGTGGACTTCACTGC	AAGGTGAAGAAGGGGTGCTC	1
NM_002748	MAPK6	mitogen-activated protein kinase 6	TGGATGAAACTCACAGTCACATT	GGCCAATCATGCTCTGAAA	48
NM_005940	MMP11	matrix metallopeptidase 11 (stromelysin 3)	AAGAGGTTCGTGCTTTCTGG	CCATGGGAACCGAAGGAT	14
NM_004994	MMP9	matrix metallopeptidase 9 (gelatinase B, 92kDa gelatinase, 92kDa type IV collagenase)	ATCCGGCACCTCTATGGTC	CAGACCGTCGGGGGAG	77
NM_001012241	MSL1	male-specific lethal 1 homolog (Drosophila)	CAGGCCAAGGAAAAGGAGAT	CGTTCAATCCGAGCAAGG	17
NM_198175	NME1	non-metastatic cells 1, protein (NM23A)	CCTAAGCAGCTGGAAGGAAC	CGCTTGATAATCTCTCCCACA	100
NM_138711	PPARG	peroxisome proliferator-activated receptor gamma	GACAGGAAAGACAACAGACAAATC	GGGGTGATGTGTTTGAACTTG	7
NM_004878	PTGES	prostaglandin E synthase	AGAAGGCCTTTGCCAACC	GATGGTCTCCATGTCGTTCC	122
NM_000963	PTGS2	prostaglandin-endoperoxide synthase 2 (prostaglandin G/H synthase and cyclooxygenase)	CGCTCAGCCATACAGCAA	TCATACATACACCTCGGTTTTGA	150
NM_175744	RHOC	ras homolog gene family, member C	AGCACACCAGGAGAGAGCTG	GTAGCCAAAGGCACTGATCC	92
NM_002964	S100A8	S100 calcium binding protein A8	CAGCTGTCTTTCAGAAGACCTG	CTTTCTCCAGCTCGGTCAAC	105
NM_001145645	TNFSF13B	tumor necrosis factor (ligand) superfamily, member 13b	CTCAAGACTGCTTGCAACTGA	AAGCTGAGAAGCCATGGAAC	112
NM_001113202	NACA*	nascent polypeptide-associated complex alpha subunit	TGCTACAGAGCAGGAGTTGC	TCCTGTTCTTCAAGCTCTGGT	45
NM_001002	RPLP0*	ribosomal protein, large, P0	TCGACAATGGCAGCATCTAC	GCCAATCTGCAGACAGACAC	6
NM_003295	TPT1*	tumor protein, translationally-controlled 1	CAATCAAAGGGAAACTTGAAGAA	GATTCATGTTTTCACCAATAAAGAAC	54
